# Multimodal evaluation of osteosarcoma choroidal metastasis

**DOI:** 10.1177/11206721221123880

**Published:** 2022-09-04

**Authors:** Federico M. Fernández Mancebo, M. Eugenia Inga, Malena Daich Varela

**Affiliations:** 1 Hospital Oftalmológico Santa Lucía, Buenos Aires, Argentina; 2 4919 Universidad de Buenos Aires (UBA), Buenos Aires, Argentina; 3 4960 Moorfields Eye Hospital, London, UK; 4 UCL Institute of Ophthalmology, 28196 University College London, London, UK

**Keywords:** Uveal tumors < TUMORS, TUMORS, uveal tumors < UVEA, retinal detachment < RETINA, techniques of retinal examination < RETINA

## Abstract

**Background:**

Osteosarcoma (OS) is the most common primary bone carcinoma. Adulthood most frequent intraocular malignant tumor is choroidal metastasis; however, these are rarely related to sarcomas. There are only two OS-related choroidal metastasis cases reported in the literature, both prior to 1970.

**Case presentation:**

A 20-year-old man with a history of tibial OS, right leg amputation, and lung and brain metastases, presented with decreased vision in his right eye (OD). Ophthalmic examination revealed a best-corrected visual acuity of hand movements and a large, posterior pole, nodular, subretinal mass, with associated fluid. B-scan revealed a heterogeneous lump, with medium/high reflectivity, and a height-to-base ratio (HBR) of 1-1.2, approximately. Computerized tomography (CT) scan showed a hyperdense and contrast-enhanced mass, while on magnetic resonance imaging (MRI) the lesion appeared T1-isointense and T2-hypointense.

**Conclusion:**

Choroidal OS metastasis can appear as a pink nodule with high HBR and intralesional hyperreflective deposits. Sudden visual changes in individuals with OS-related systemic metastatic disease should be monitored closely by ophthalmology and oncology jointly.

## Introduction

Osteosarcoma (OS) is a mesenchymal malignant neoplasm in which cancerous cells synthesize and secrete bone matrix components.^1^ It is the most common primary bone malignancy, with a bimodal age distribution (adolescents and elderly), and an approximate incidence of 4/million/year worldwide.^2^ Metastatic disease is frequent in OS, often leading to treatment failure and palliative care.^3^ Lungs are usually affected,^4^ while ocular involvement is rare. To the best of our knowledge, there are only two cases of OS-related choroidal metastasis reported in the literature; one published in 1947 and the other in 1968.^5,6^ In this report, we present the first twenty-first century multimodal evaluation of a young man with choroidal OS metastasis.

## Case report

**Figure 1.**
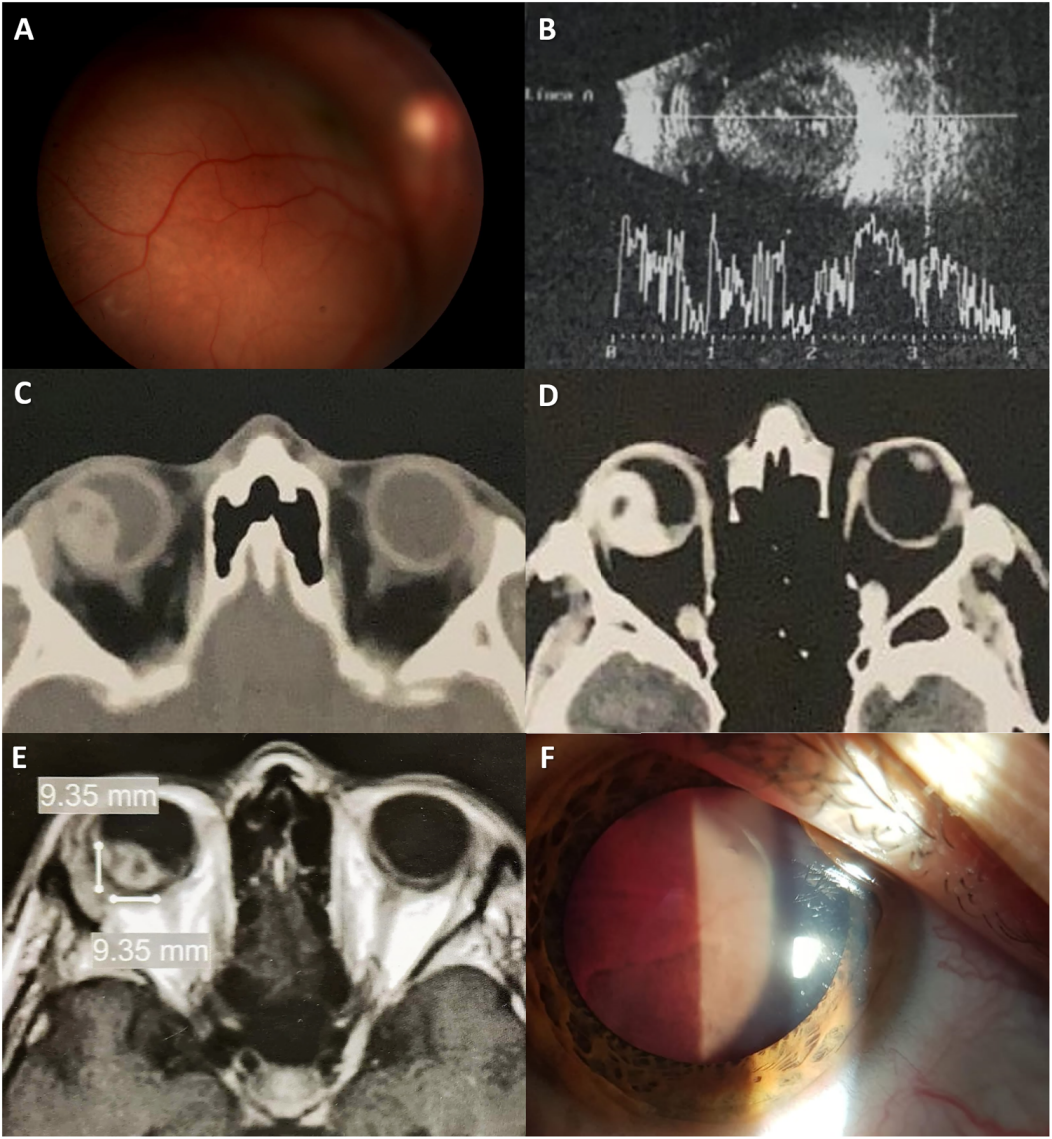
Multimodal evaluation of osteosarcoma choroidal metastasis. A) Right eye (OD) retinography, showing an elevated subretinal, pink, nodular mass on the posterior pole. B) A and B-scan OD ultrasound positive for a large, round, subretinal mass with irregular structure, medium/high overall reflectivity and areas of localized increased and decreased (probably necrosis-related air) reflectivity, with a height-to-base ratio of 1-1.2, approximately. C) Computerized tomography (CT) scan revealing a heterogeneous, well-defined mass within the uveal layer, hyperdense with areas of decreased density, and significant intraocular involvement. D) Contrast-enhanced CT showing a heterogeneously enhancing mass on OD, with areas of decreased and null enhancement. E) T1- weighted magnetic resonance imaging showing a well-defined, heterogeneous choroidal mass that appears isointense and has equal base and height. F) Anterior segment photography taken at a follow up visit showing a fully detached retina in contact with the posterior capsule of the lens.

A 20-year-old man and his family were involved in this study. The parents provided a written consent to participate in the present study (which took place after the patient's death), adhering to the tenets of the Declaration of Helsinki. This young man presented to the ophthalmology emergency department due to decreased vision on his right eye (OD). His visual acuity had dropped two weeks prior to the consult, without further worsening. He had a history of right tibial osteosarcoma diagnosed at age 17, treated with supracondylar amputation, and a recent diagnosis of brain and lung metastases. Histopathology was available for the tibial lesion only; while secondary tumors were diagnosed by the oncologist through multimodal imaging. Ophthalmic examination revealed a best-corrected visual acuity of hand movements OD and 20/20 on the left eye. The anterior segment was unremarkable bilaterally and the posterior segment of OD had a large, posterior pole, pink, nodular, subretinal mass, with associated fluid (Figure 1A). Left posterior segment exam was normal. An A and B-scan ultrasonography confirmed that the mass was subretinal, heterogeneous, with medium/high reflectivity, areas of localized increased and decreased (probably necrosis-related air) reflectivity, and a height-to-base ratio (HBR) of 1-1.2, approximately (Figure 1B). Optical coherence tomography was attempted but due to the mass' large dimensions, it was not possible to get a complete scan. A subsequent computerized tomography (CT) revealed a heterogeneous, well-defined mass within the uveal layer, hyperdense, with areas of decreased density, significant intraocular involvement, and contrast-enhancement (Figure 1C and D). In addition, magnetic resonance imaging (MRI) showed a well-defined, irregular choroidal mass, T1-isointense (Figure 1E) and T2-hypointense. The history of widespread disease and the characteristics of the lesion were compatible with choroidal OS metastasis. His oncologist was notified and together with the family, the decision was to adjust his chemotherapy treatment and observe the ocular lesion.

At a one-month follow up assessment, OD was painful, had a fully detached retina, anterior segment neovascularization, and an intraocular pressure of 38 mmHg (Figure 1F). The patient only agreed to topical cycloplegic treatment, with mild symptom improvement. During follow up, the ocular lesion responded to the systemic treatment and decreased in volume, according to CT and MRI scans. Lung and brain metastases were not as responsive however, and the patient passed away one year after the first ophthalmologic assessment due to systemic complications.

## Conclusions

The choroid is the most commonly affected ophthalmic region for metastatic disease, due to its high vascularity. Choroidal metastases account for 2 to 7% of all secondary lesions.^7^ The primary tumors that most commonly metastasize to the choroid are breast and lung cancer, while sarcomas spreading to this tissue are very rare.^8^ The diagnosis of choroidal metastasis is primarily clinical, currently aided by multimodal imaging studies. Fine needle aspiration biopsy may also be useful in cases of unclear primary tumor or diagnosis.^8^ Choroidal metastases frequently occur in the later stages of disseminated disease and are considered a poor prognostic sign.

Certain particularities regarding color and shape of choroidal secondary lesions have been proposed, associating lung carcinoma with pale-yellow color; breast cancer with creamy-white, lobular shaped, multifocal, and diffuse forms; and renal, thyroid and carcinoid tumors with orange color.^7^ These are all typically flat or slightly dome-shaped, with an HBR below 0.2. OS metastasis can appear pink, with a nodular/round shape, and high HBR (closer to choroidal melanoma values rather than metastasis ones).^5,7^ OS B-scans can appear with hyperreflective deposits and, similarly to other metastases, medium/high heterogeneous reflectivity; in this case possibly due to the trabecular pattern of osteoid tissue. CT scan and MRI characteristics were similar to other choroidal metastases.^9^ Interestingly, the fundus imaging and presentation of our patient resembles a chondrosarcoma choroidal metastasis case, with both patients presenting with a large, single, nodular, amelanotic lesion, with associated subretinal fluid.^10^ This points to possible common features shared by bone sarcomas metastasizing to the eye.

Metastatic disease is the main cause of mortality in individuals with OS.^4^ Although ocular involvement is rare, it can affect the orbit, eyelid and choroid, and both oncologists and ophthalmologists should bear this in mind. We present different ways in which intraocular OS metastasis can be assessed, and its B-scan, CT and MRI features. Timely diagnosis can lead to early interventions (chemotherapy adjustment, immunotherapy, or radiotherapy)^8^ that may improve quality of life.
